# The Relationship between Population T4/TSH Set Point Data and T4/TSH Physiology

**DOI:** 10.1155/2016/6351473

**Published:** 2016-03-31

**Authors:** Stephen Paul Fitzgerald, Nigel Geoffrey Bean

**Affiliations:** ^1^Department of Internal Medicine and Department of Endocrinology, The Royal Adelaide Hospital, Adelaide, SA 5000, Australia; ^2^School of Medicine, The University of Adelaide, Adelaide, SA 5005, Australia; ^3^School of Mathematical Sciences, The University of Adelaide, Adelaide, SA 5005, Australia; ^4^ARC Centre of Excellence for Mathematical and Statistical Frontiers, The University of Adelaide, Adelaide, SA 5005, Australia

## Abstract

*Context*. Population studies of the distribution of T4/TSH set points suggest a more complex inverse relationship between T4 and TSH than that suggested by physiological studies. The reasons for the similarities and differences between the curves describing these relationships are unresolved.* Methods*. We subjected the curve, derived from empiric data, describing the TSH suppression response to T4, and the more mathematically derived curve describing the T4 response to TSH, to the different possible models of population variation. The implied consequences of these in terms of generating a population distribution of T4/TSH equilibrium points (a “population curve”) were generated and compared to the empiric population curve. The physiological responses to primary hypothyroidism and hyperthyroidism were incorporated into the analysis.* Conclusions*. Though the population curve shows a similarly inverse relationship, it is describing a different relationship than the curve describing the suppression of TSH by T4. The population curve is consistent with the physiological studies of the TSH response to T4 and implies a greater interindividual variation in the positive thyroid T4 response to TSH than in the central inhibitory TSH response to T4. The population curve in the dysthyroid states is consistent with known physiological responses to these states.

## 1. Introduction

**Figure 1:**
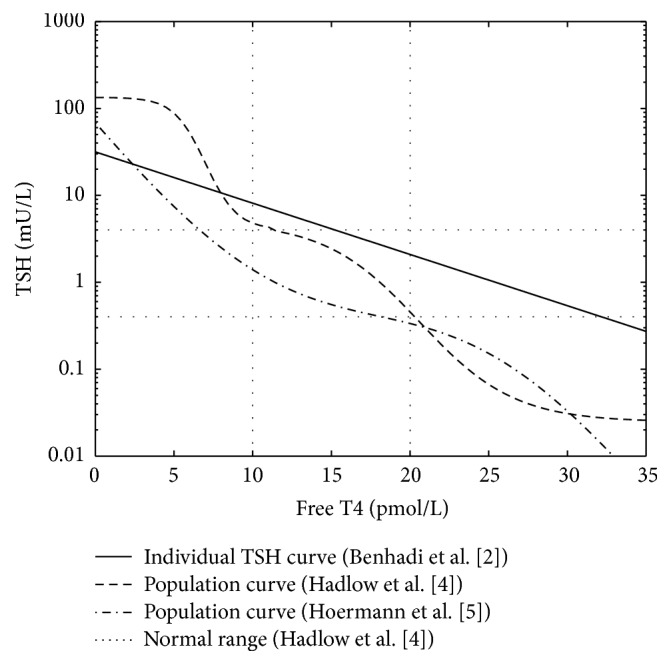
Comparison of 2 representations of the population distribution of T4/TSH (the population curve) with the T4/TSH physiological relationship as described in individuals (the TSH curve).

**Figure 2:**
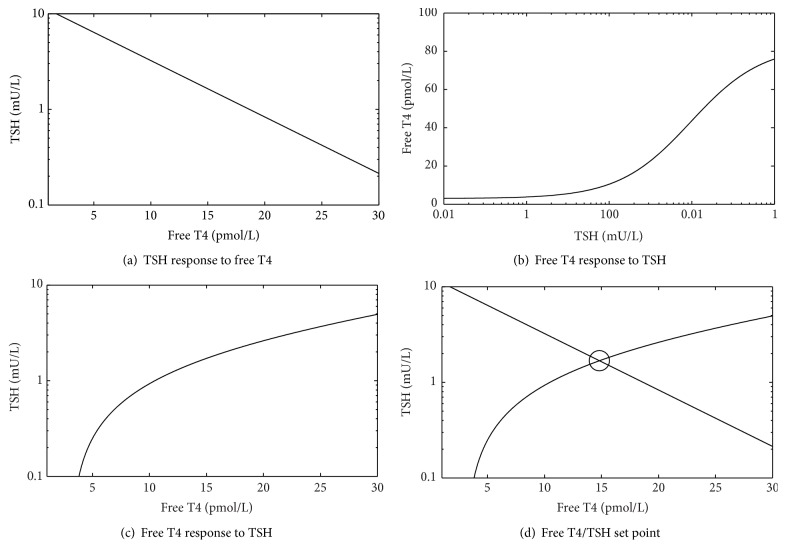
(a) The TSH curve, (b, c) the T4 curve (on different axes, *K*_T_ approx. 41), and (d) the location of the T4/TSH set point.

There has been debate in the literature as to the reconciliation of the relationships between T4 and TSH. Whilst the curve describing the suppression of TSH by circulating T4 as studied in individuals (the “TSH curve”) is negative log-linear [1–3], population distributions of set point T4/TSH levels in multiple individuals (“the population curve”) have suggested a somewhat similar but more complex negative relationship (Figures 1 and 2(a)) [4, 5]. The TSH curve has been determined by T4 dosing studies whereby TSH levels were measured in individuals at different levels of T4 [1–3]. The population curve is cross-sectional and results from the plotting of simultaneous T4 and TSH (the set point) measurements of a large number of individuals [4, 5].

In particular the curves differ in that the population curve is flatter at the extremes but otherwise has increased slope in the hypothyroid and hyperthyroid ranges [4]. The flattening at the extremes has been attributed to the limits of physiological responses [4, 6]; the other differences between the curves remain unresolved.

Different explanations invoking different artefacts of measurement and population have been offered for the discrepancies between the above curves [4, 5]. It has been suggested that the population curve indicates that the negative log-linear relationship is incomplete or erroneous [4, 5, 7], and more complicated models of thyroid regulation have been suggested [7–10].

The reciprocal physiological T4/TSH relationship, the transmission of TSH to T4, whereby circulating TSH stimulates T4 levels (the “T4 curve”), is more difficult to define experimentally. Its general properties have been confirmed experimentally [11–13] and clinically [14] and on the basis of these data and pharmacological principles [15] have been defined mathematically (Figures 2(b) and 2(c)) [7, 16]. We term the TSH and T4 curves the “physiological curves.” Though individual set points have been recognised to be situated at the intersection of the two physiological curves [7, 16] (Figure 2(d)), the formulation of any relationship between the population curve and interindividual variations in these two curves has not been pursued.

The aim of this study was to reconcile the general slope of the population curve with the physiological curves by considering the effects of interindividual variations in these physiological curves. Furthermore we sought to explain the curvature of the population curve in terms of changes to the physiological curves due to previously described physiological responses to primary hypo- and hyperthyroidism.

## 2. Methods

No new empiric data were generated. In describing the T4/TSH relationships we considered T4 and FT4 to be interchangeable terms describing free T4. We considered pituitary function to include hypothalamic physiology.

We relied on the empiric data already available from the literature for the population curve (Figure 1) and the TSH curve (Figures 1 and 2(a)). Benhadi et al.'s [2] line of best fit for the TSH curve, typical for this relationship, is described by the formula log⁡TSH = 1.50 − 0.059FT4. The line of best fit for the population curve has been described in at least two ways. Hoermann et al. [5] considered the curve was best described in terms of response varying in relation to the “error” function, that is, the disturbance in T4 away from an optimum value. Their formula was log⁡TSH = *π* ^0.5^erf(0.09(FT4 − 18)) − 0.22(FT4 − 18) − 0.39, where erf(·) is the error function. In the T4 range 12–16 this resulted in a line not dissimilar to TSH = −0.6FT4 + 0.05. Hadlow et al. [4] considered the curve to be a composite of two sigmoid curves. For free T4 concentrations ≤12 the relationship was ln⁡TSH = 1.4 + 3.5/(1 + *e* ^−(7.0−FT4)/1.0^); for free T4 concentrations >12 it was ln⁡TSH = −3.7 + 5.3/(1 + *e* ^−(20.6−FT4)/3.0^).

We used the thyroid transfer characteristic (the “T4 curve”) (Figures 2(b) and 2(c)) as described by Dietrich et al. [7] and Goede et al. [16]. Goede et al. [16] have indicated that this relationship is described by the formula [FT4] = *K*_T_[TSH]/(*D*_T_ + [TSH]) pmol/L. *K*_T_ represents the maximum T4 yield, and *D*_T_ represents the damping constant (EC50) of TSH at the thyroid gland, that is, the concentration of TSH that gives half-maximal T4 response. This formula is “based on Michaelis-Menten kinetics as they are well grounded in physiological and biochemical grounds…” [16].

We continued the line of this previous work [7, 16] by considering that different individuals, by virtue of differing organ size, and/or differences in any of a myriad of thyroid physiological processes (e.g., circulating T3 level and intrapituitary deiodination), will have greater or lesser increases in T4 secondary to TSH stimulation and analogously greater or lesser TSH suppression by T4. These variations result in different individuals having different T4 and TSH curves.

**Figure 3:**
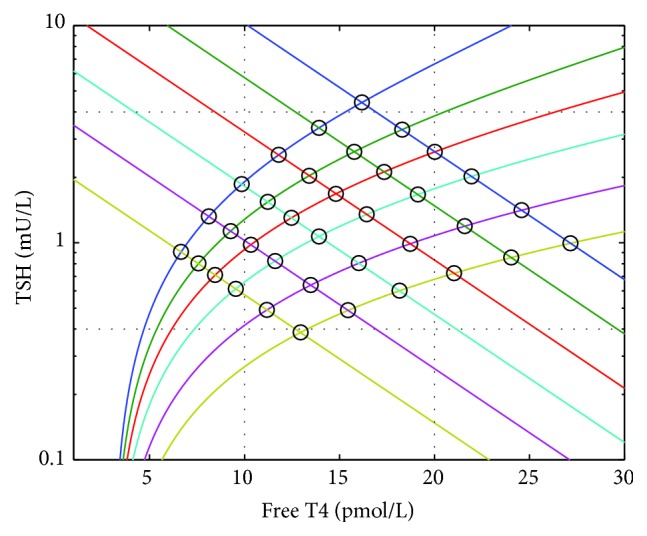
The generation of the T4/TSH set points in a population. The circles represent the different potential set points of different individuals (generated as per Figure 2) that are lying on the intersection points of their individual T4 and TSH curves.

The T4/TSH equilibrium point was represented by the intersection of the physiological curves as described by Dietrich et al. [7] and Goede et al. [16] (Figure 2(d)). Dietrich et al. [7] indicated a set point lying on the intersection of the two 50th centile curves but we extended this model to indicate that a range of set points is generated by the intersection points of each individual's two curves. This range of set points in the population is thus the basis of the population curve (Figure 3).

We generated graphs to describe different possible population curves as generated by the possible different patterns of interindividual variation in the two physiological curves, that is, variation in the TSH curve being greater than, less than, or equal to variation in the T4 curve.

We compared the general slope of our resulting curves with that of the published empirically derived population curve.

We also factored into our graphs the previously documented physiological changes to the pituitary in the contexts of primary hypo- and hyperthyroidism to determine whether these physiological changes generated curvatures consistent with those in the published empiric curve.

## 3. Results

**Figure 4:**
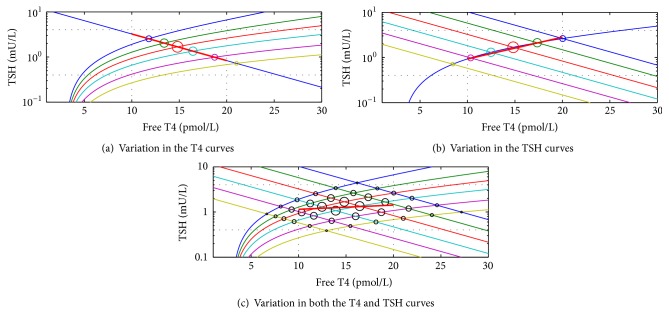
(a) shows a population in which interindividual T4 curve variation is greater than interindividual TSH curve variation (the extreme example of only one TSH curve intersecting with multiple T4 curves is shown), (b) shows the converse, one T4 curve intersecting with multiple TSH curves, and (c) shows a population in which T4 and TSH curve variation are similar (there are multiple T4 and multiple TSH curves and more intersection points). In these graphs the intersection points are marked by circles of different sizes to indicate a typical distribution of the curves and the larger circles indicate that more individuals are expected to lie in the centre of the range as compared to the extremities. The lines of best fit (thick red lines) are drawn such that the differences in the possible slopes of the lines of best fit (the different population curves) are apparent.

Figure 4(a) shows how the population curve would appear if there were relatively little or no population variations in the TSH curve but there was a large variation in the T4 curve; it would resemble the TSH curve (a negative slope). In these circumstances interindividual T4 level variation is predominantly determined by the T4 curves.

Figure 4(b) shows how the population curve would appear if conversely there were relatively little or no population variations in the T4 curve but there was a large variation in the TSH curve, and thereby the predominant determinant of the T4 level variation is the individual TSH curves (a positive slope).

Figure 4(c) shows how the population curve would appear if there were equal variations in both pituitary and thyroid sensitivities (no strong relationship). In this latter circumstance the TSH and T4 curves would contribute equally to the interindividual variation of the T4 level.

**Figure 5:**
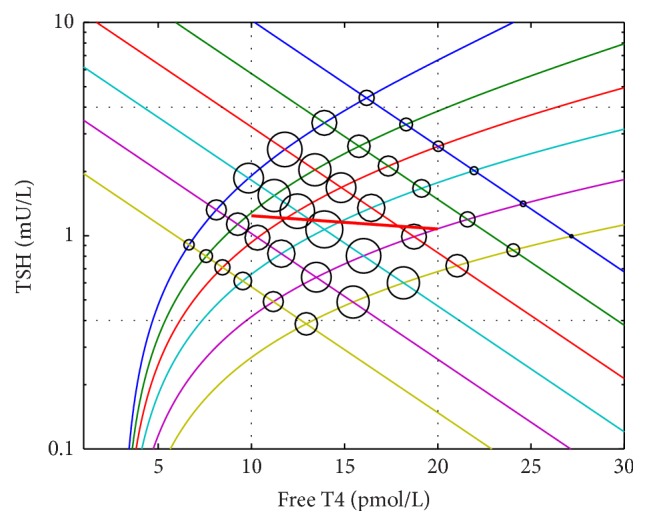
The generation of a population T4/TSH relationship by nonrandom associations between the physiological curves. In this example there is a hypothetical association between insensitive thyroids and sensitive pituitaries (this might occur by evolution to minimise T4 variation). The sizes of the circles, as in Figure 4, are proportionate to the number of individuals in the population with T4/TSH levels at that point. The line of best fit has a small negative slope.

These relationships are as described above under the assumption that the physiological curves of all individuals are independent of each other. In the population context of multiple physiological T4 and TSH curves the population curve would also depend on associations between the two physiological curves. If, for example, some aspects of physiology or natural selection led to nonrandom/nonproportional associations of the physiological curves, the slope of the line of best fit of the population curve might lie anywhere within the bounds of the population variations of the physiological curves or a strong relationship may arise where, in the context of independent curves, there was not any (Figure 5).

Thus the general slope of the population curve may resemble any of the curves of Figure 4 depending on the nature of the interindividual variation in each of the physiological curves. It follows that the population curve cannot of itself describe the nature of the TSH curve and in particular cannot deny the evidence of studies of the physiology of individuals.

Given that in having a negative slope the empirically derived population curve (Figure 1) resembles Figure 4(a), or perhaps Figure 5 in part of the normal range, one can deduce from all the above that the negative slope of the population curve results from the greater variation in thyroid as compared with hypothalamo-pituitary sensitivity, and/or the effects of associations between the curves. The slope (−0.6) of the population curve in the normal range [5] is similar to the slope (−0.59) of the curve of physiological TSH studies [2], and therefore it would seem more likely that the slope there has been generated by the mechanism described in Figure 4(a), that is, the different degrees of variation in organ sensitivity.

We found that the increases in slope moving away from the edges of the normal T4 range [4] are consistent with adaptive shifts in the TSH curve in response to low and high T4 levels. The slope of the different individuals' TSH response curves need not necessarily change. These shifts are consistent with thyrotroph hyperplasia and hypertrophy in primary hypothyroidism and thyrotroph atrophy and degeneration in hyperthyroidism [7, 17, 18] as well as other possible physiological changes [7, 10].

**Figure 6:**
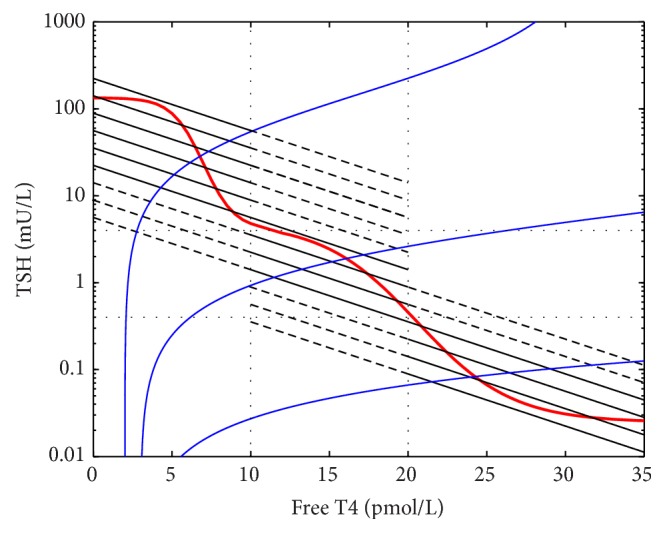
The effect of vertical shifts in the TSH curves, induced by primary thyroid dysfunction, on the points of intersection of the physiological curves. On account of these changes the T4 curves in the hypothyroid and hyperthyroid ranges do not intersect with the extensions of the TSH curves of the normal range (these extensions are shown as dashed lines) and therefore the slope of the population curve increases. The extensions of the TSH curves into the normal range from the regions of thyroid dysfunction are also shown as dashed lines to indicate that they do not normally exist in this range. The red line is the population curve as per Hadlow et al. [4].

Therefore outside of the normal range because the TSH response curves are shifted vertically any points of intersection with the T4 response curves are also so shifted; that is, with progressive hypothyroidism/hyperthyroidism the points of intersection are on progressively changing TSH curves (Figure 6). Thus the population curve incorporates changes in organ sensitivity over time in response to ambient physiology whereas the physiological curves are based on studies that have been conducted over a relatively short period of time and therefore with stable organ sensitivities.

## 4. Discussion

Though thyroid physiology and in particular the physiology of the control of thyroid hormones is complex, it can be simplified to this model based on the two physiological curves [16]. Though T3, the active thyroid hormone [19], acting via the TR*β*2 receptor, and not T4, is regarded as the regulator of TSH physiology and gene expression [20–22], and though there is also a negative log-linear relationship between T3 and TSH [2], Goede et al. [16] point out that the model does not require consideration of T3 levels. They state, “Using a model with two degrees of freedom allows the contributory factor exerted by [FT3] to be completely subsumed within the two structural parameters such that only [FT4] remains the stimulus variable connecting the [TSH] as the response.”

The pituitary, though ultimately responsive to T3, is more responsive to T3 generated in the pituitary from circulating T4 by type 2 deiodinase than to circulating T3, and TSH levels are more consistently related to levels of T4 than T3 [7, 23–25]. There are physiological advantages of this preference [7].

Various other components of thyroid physiology contribute to the derivation of, and are embedded in, the physiological curves, but as such, their detailed consideration is also not material to this discussion. Such components include deiodinases, hormone transporters, transcription factors, and other processes of thyroid hormone metabolism. All of these other processes of thyroid physiology influence the derivation of these two curves in any individual and thus the interindividual differences.

There has been confusion regarding the information to be derived from population studies of the T4/TSH set point. There are a few possible reasons for this. The similarity of the population curve and the TSH curve may have led researchers [4, 5, 7, 8] to believe that the two curves describe the same physiology. Differences between the curves have led to discussion as to which curve better describes this physiology [4, 5, 7–9].

The physiological studies of individuals show this inverse relationship between T4 and TSH because of the method of study; that is, in individuals the TSH was measured at different levels of T4 [1–3]. The other, equally important curve of measured T4 with different levels of TSH is more difficult to define empirically and perhaps on account of this, and perhaps because of the believed primacy of hypothalamo-pituitary function [26], there has been no discussion in the above literature as to why the population curve does or does not resemble the T4 curve.

The fact that the population graph has a negative slope reminiscent of the TSH curve can most simply be accounted for by a greater population variation in thyroid sensitivity to TSH as compared with pituitary sensitivity to T4. Outside of the normal range the relationship is consistent with the greater preponderance of primary as distinct from secondary thyroid dysfunction [7, 27, 28]. This phenomenon has been exaggerated by the methodology of the population studies whereby those individuals with a suggestion of pituitary dysfunction were excluded [4, 5, 10]. A less parsimonious explanation might invoke associations between the two physiological curves. Still however the population curve would reflect the pattern of interindividual variation in the T4 and TSH curves.

Our results are robust as to the shape of the physiological curves and were identical when we considered different formulas for the different curves [2, 4, 5, 29]. Similarly our results do not depend upon the nature of variation in the physiological curves (e.g., the different TSH curves need not be parallel). Our results will apply so long as there is, as is implied by the feedback loop physiology, a physiologic range whereby T4 levels increase in response to increasing TSH levels and TSH levels decrease in response to increasing T4 levels.

Leow [8] has indicated that each individual on the population curve may have a TSH curve consistent with a log-linear relationship and that the TSH curve may vary in different functional states. Midgley et al. [10] have also discussed differing physiology in the various thyroid states contributing to the various slopes of different segments of the population curve. These authors do not however demonstrate that the slope of the population curve is dependent on how the variation of the T4 curve compares with the variation of the TSH curve and that this slope is subject to modulation by adaptive changes to the TSH curve in the hyper- and hypothyroid ranges.

Our findings regarding the population curve in the hypo- and hyperthyroid ranges extend previous work. Such studies of the TSH curve have indeed shown similarly straight curves for hypothyroid [1, 2] and euthyroid [2] individuals. The shift between them has been attributed to the treatment of hypothyroidism with T4 and a consequent relative deficiency of T3 [1]. This in turn was interpreted as demonstrating that T3 provides the dominant control over TSH levels even though the study itself was not entirely consistent with this proposition and despite other studies [24] (confirmed since [23]) that there is not a good relationship between T3 and TSH levels. Furthermore one of the recent population studies showed that the relationship between T4 and TSH appeared to be similar in subjects receiving thyroxine therapy and untreated individuals at steady euthyroid state [4]. All of this data is consistent with T4 being the dominant controller of TSH levels [25], and the shift in the TSH curve in hypothyroid individuals being rather the result of the physiological changes to the pituitary and hypothalamus in hypothyroidism. The population curve, relying on the intersection of the T4 curves with these shifted TSH curves, is consistent with the effects of these changes.

Other aspects of physiology [10], pathophysiology [30] and technical factors regarding the assays [31] might also influence the population curve and its scatter. The changes in the population curve depending on age and sex [4] are also explicable by changes related to age and sex to the two physiological curves [32, 33].

Our proposed relationship between the population curve and the T4 and TSH curves is amenable to further study and disproof. The contributions of variations in organ sensitivity, physiological associations, hypertrophy, atrophy, and so forth could be elucidated, for example, by mapping the individual physiological curves contributing to individual population points and by studying in humans or animals any change in the physiological curves over time in response to changes in T4 levels. The difficulty in mapping T4 curves presents a technical challenge.

Our work complements the previous work [10, 34, 35] on computer simulation of thyroid regulation and may contribute to set point theory.

## 5. Conclusion

In summary, this work offers a resynthesis of the empiric data concerning the T4/TSH relationship, demonstrating that the previously derived individual physiological data and population set point data refer to different relationships, which are compatible with each other, and that therefore the former needs no revision on account of the latter. The similarities and differences previously noted are readily explained by population variations in physiology and by the known physiological responses to primary thyroid dysfunction. In turn the population data imply a particular pattern of interindividual variation of thyroid and pituitary physiology. This clarification and simplification of the T4/TSH relationship, apart from having intrinsic value, may contribute to the further understanding of thyroid physiology and in particular the understanding of thyroid regulation.
