# Corrigendum to “The Relationship between Population T4/TSH Set Point Data and T4/TSH Physiology”

**DOI:** 10.1155/2017/6917841

**Published:** 2017-08-20

**Authors:** Stephen Paul Fitzgerald, Nigel Geoffrey Bean

**Affiliations:** ^1^Department of Internal Medicine and Department of Endocrinology, The Royal Adelaide Hospital, Adelaide, SA 5000, Australia; ^2^School of Medicine, The University of Adelaide, Adelaide, SA 5005, Australia; ^3^School of Mathematical Sciences, The University of Adelaide, Adelaide, SA 5005, Australia; ^4^ARC Centre of Excellence for Mathematical and Statistical Frontiers, The University of Adelaide, Adelaide, SA 5005, Australia

In the article titled “The Relationship between Population T4/TSH Set Point Data and T4/TSH Physiology,” [1] there was an error in Figure 2(b), which should be corrected as shown in Figure 2.

Additionally, there were errors in Results, where the statement “The slope (−0.6) of the population curve in the normal range [5] is similar to the slope (−0.59) of the curve of physiological TSH studies [2], and therefore it would seem more likely that the slope there has been generated by the mechanism described in Figure 4(a), that is, the different degrees of variation in organ sensitivity” should be corrected to “The slope (−0.06) of the population curve in the normal range [5] is similar to the slope (−0.059) of the curve of physiological TSH studies [2], and therefore it would seem more likely that the slope there has been generated by the mechanism described in Figure 4(a), that is, the different degrees of variation in organ sensitivity.”

## Figures and Tables

**Figure 2:**
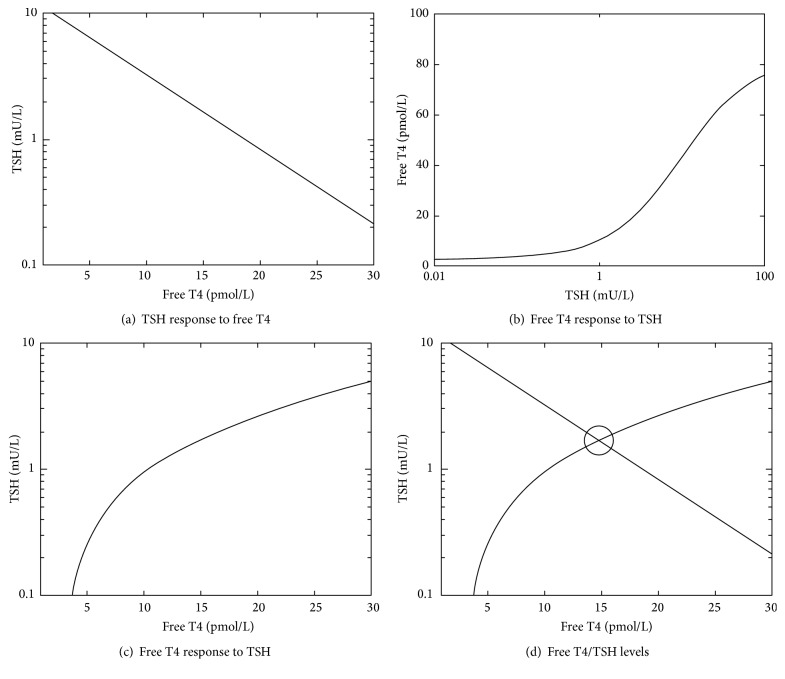
(a) The TSH curve, (b, c) the T4 curve (on different axes, *K*_T_ approx. 41), and (d) the location of the T4/TSH set point.
